# Molecular basis for the dosing time-dependency of anti-allodynic effects of gabapentin in a mouse model of neuropathic pain

**DOI:** 10.1186/1744-8069-6-83

**Published:** 2010-11-26

**Authors:** Naoki Kusunose, Satoru Koyanagi, Kengo Hamamura, Naoya Matsunaga, Miyako Yoshida, Takahiro Uchida, Makoto Tsuda, Kazuhide Inoue, Shigehiro Ohdo

**Affiliations:** 1 Department of Pharmaceutics, Graduate School of Pharmaceutical Sciences, Kyushu University, Fukuoka, Japan; 2 Department of Molecular and System Pharmacology, Graduate School of Pharmaceutical Sciences, Kyushu University, Fukuoka, Japan; 3 Department of Clinical Pharmaceutics, Faculty of Pharmaceutical Sciences, Mukogawa Women's University, Hyogo, Japan

## Abstract

**Background:**

Neuropathic pain is characterized by hypersensitivity to innocuous stimuli (tactile allodynia) that is nearly always resistant to NSAIDs or even opioids. Gabapentin, a GABA analogue, was originally developed to treat epilepsy. Accumulating clinical evidence supports the effectiveness of this drug for diverse neuropathic pain. In this study, we showed that the anti-allodynic effect of gabapentin was changed by the circadian oscillation in the expression of its target molecule, the calcium channel α2δ-1 subunit.

**Results:**

Mice were underwent partial sciatic nerve ligation (PSL) to create a model of neuropathic pain. The paw withdrawal threshold (PWT) in PSL mice significantly decreased and fluctuated with a period length about 24 h. The PWT in PSL mice was dose-dependently increased by intraperitoneal injection of gabapentin, but the anti-allodynic effects varied according to its dosing time. The protein levels of α2δ-1 subunit were up-regulated in the DRG of PSL mice, but the protein levels oscillated in a circadian time-dependent manner. The time-dependent oscillation of α2δ-1 subunit protein correlated with fluctuations in the maximal binding capacity of gabapentin. The anti-allodynic effect of gabapentin was attenuated at the times of the day when α2δ-1 subunit protein was abundant.

**Conclusions:**

These findings suggest that the dosing time-dependent difference in the anti-allodynic effects of gabapentin is attributable to the circadian oscillation of α2δ-1 subunit expression in the DRG and indicate that the optimizing its dosing schedule helps to achieve rational pharmacotherapy for neuropathic pain.

## Background

Neuropathic pain is a chronic condition that occurs after bone compression in cancer, diabetes, herpesvirus infection and auto immune disease [1]. Millions of patients in the world presently endure neuropathic pain [2]. One troublesome hallmark symptom of neuropathic pain is hypersensitivity to normally innocuous stimuli, a condition known as "tactile allodynia" that is often refractory to NSAIDs and opioids [3].

The GABA analogue gabapentin was originally developed to treat epilepsy [4], but it is now widely used to alleviate neuropathic pain [3]. Accumulating evidence from diverse animal models of neuropathic pain suggests that the anti-allodynic effects of gabapentin are associated with the modulation of neurotransmitter release or neuronal excitability resulting from alterations in Ca^2+ ^currents [5,6]. The α2δ subunit, but not the pore-forming α1 or β subunits, of voltage-dependent Ca^2+ ^channels (VDCC) in the spinal cord and dorsal root ganglion (DRG) is upregulated in gabapentin-sensitive pain models such as mechanical- and diabetic-neuropathic types, but not in the gabapentin-insensitive chemical model of neuropathic pain [7-9]. The time course of upregulation of the α2δ subunit in DRG is parallel to the duration of neuropathic pain induced by nerve injury [9]. Gabapentin is thought to modulate Ca^2+ ^currents by binding to the α2δ-1 subunit of VDCC [10-12].

Gabapentin is now widely used to alleviate neuropathic pain because it is well-tolerated, easily titrated, and has interacts with few drugs [13]. However, higher doses of gabapentin can cause side effects such as dizziness, drowsiness, and peripheral edema [14]. The appropriate dosing schedule of gabapentin has not been well-established yet.

One approach to increasing the effect of pharmacotherapy is to administer drugs at the times of day when they are most effective and/or best tolerated. Circadian variations in biological functions such as gene expression and protein synthesis are thought to be important factors affecting the efficacy of drugs. In fact, the expression levels of proteins related to the regulation of the susceptibility to the drugs and their pharmacokinetics oscillated in a circadian time-dependent manner [15-17]. It is thus possible that the pharmacological effects of gabapentin could be more improved by choosing the appropriate dosing time. To test this possibility, we explored whether the anti-allodynic effects of gabapentin in a mouse model of neuropathic pain was changed according to its dosing time. The mechanism underlying dosing time-dependent changes in the anti-allodynic effects of gabapentin was investigated from the perspectives of pharmacokinetics and pharmacodynamics.

## Results

### Time-dependent changes in allodynic behavior of mice

**Figure 1:**
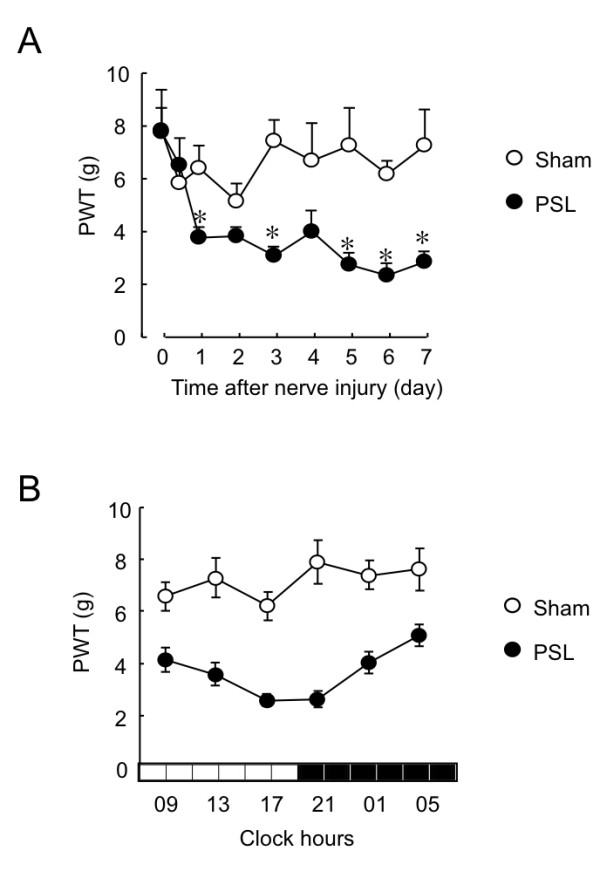
**Time-dependent variation in PWT of PSL mice**. (A) Time course of PWT of PSL and sham operated mice after nerve injury. Values are shown as means with S.E.M. (n = 6). *P < 0.05 compared with sham group at corresponding time points. (B) Variation in PWT over 24 h in PSL and sham-operated mice on day 7 after nerve injury. Values are shown as means with S.E.M. (n = 12). The PWT in PSL, but not in sham operated mice, significantly varied over 24 h (P < 0.05; ANOVA).

To create an animal model of neuropathic pain, we prepared mice undergone partial sciatic nerve ligation (PSL). As reported previously [18], the paw withdrawal threshold (PWT) of PSL mice significantly decreased after nerve injury (P < 0.05; Figure 1A). The decrease in PWT continued until at least 3 weeks (data not shown). On day 7 after nerve injury, the PWT in PSL mice significantly varied over 24 h, with trough levels persisting from the late light phase, to the early dark phase (P < 0.05; Figure 1B). The time-dependent difference of PWT of PSL mice began to be observed on day 5 after nerve injury. On the other hand, PWT in sham-operated mice did not significantly change time-dependently.

### Influence of dosing-time on anti-allodynic effects of gabapentin in PSL mice

**Figure 2:**
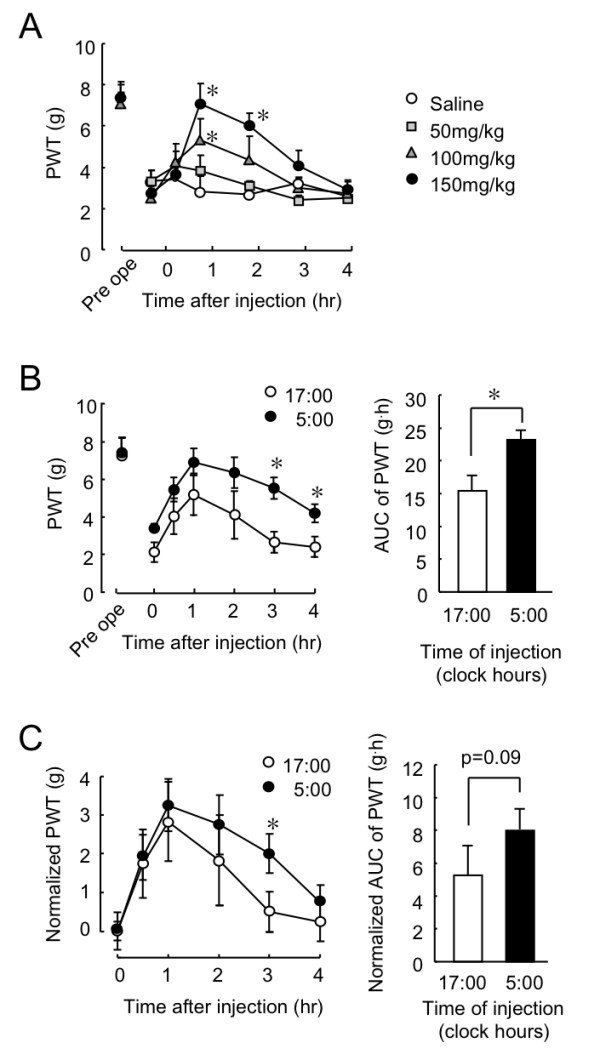
**Influence of dosing-time on the anti-allodynic effects of gabapentin in PSL mice**. (A) Time course of PWT in PSL mice after i.p. injection with 50, 100 and 150 mg/kg gabapentin or saline on day 7 after nerve injury. Values are shown as means with S.E.M. (n = 6). *P < 0.05 compared with saline-treated group. (B) Dosing time-dependence of anti-allodynic effects of gabapentin in PSL mice. Gabapentin (100 mg/kg, i.p.) was injected into PSL mice at 17:00 or 5:00. The AUC of PWT after gabapentin injection was calculated using trapezoidal rule. Values are shown as means with S.E.M. (n = 6). In left panel, *P < 0.05 compared with 17:00 group at corresponding dosing time. In right panel, *P < 0.05 compared between two groups. (C) Comparison of normalized PWT (left) and AUC (right) after gabapentin (100 mg/kg, i.p.) injection. *P < 0.05 compared with 17:00 group at corresponding dosing time.

On day 7 after nerve injury, PSL mice were injected intraperitoneally (i.p.) with 50, 100, and 150 mg/kg of gabapentin or saline at 17:00. A single injection of gabapentin transiently and dose-dependently increased the PWT of PSL mice (Figure 2A). The PWT reached a peak level around 1 h after gabapentin injection and declined to the basal level within 4 h. A significant attenuation of tactile allodynia was found at the dosage of gabapentin over 100 mg/kg.

We next investigated whether the anti-allodynic effects of gabapentin varied according to dosing time. On day 7 after nerve injury, PSL mice were injected i.p. with 100 mg/kg of gabapentin at 17:00 or 5:00. These time points were selected based on the peaks and troughs of the allodynic behavior of PSL mice when not administered with the drug (Figure 1B). Although the PWT of PSL mice was transiently increased after gabapentin injection at both time points (Figure 2B left panel), the PWT was much higher in mice injected with gabapentin at 5:00 than at 17:00. Consequently, the area under the curve (AUC) of the PWT after gabapentin injection at 5:00 was also larger than that after the drug injection at 17:00 (p < 0.05, Figure 2B right panel).

By normalizing the basal level of PWT, the anti-allodynic effect of gabapentin was also compared between the two time points. At 2, 3, and 4 h after gabapentin injection, the PWT of PSL mice were higher at 5:00 than at 17:00 (Figure 2C left panel). A significant dosing time-dependent difference in the PWT of PSL mice was observed at 3 hr after gabapentin injection (p < 0.05). Furthermore, normalized AUC of the withdrawal threshold after gabapentin injection at 5:00 tended to be significantly larger than that after the drug injection at 17:00 (p = 0.09, Figure 2C right panel). These results suggest that the anti-allodynic effect of gabapentin was varied according to its dosing time. The dosage of gabapentin more significantly alleviated tactile allodynia in PSL mice at 5:00 than at 17:00. The difference in the dosing time-dependent anti-allodynic effects of gabapentin seemed not to be associated with sedation because the mouse locomotor activities were not significantly suppressed by 100 mg/kg gabapentin (Additional file 1).

### Influence of dosing time on the gabapentin pharmacokinetics

**Figure 3:**
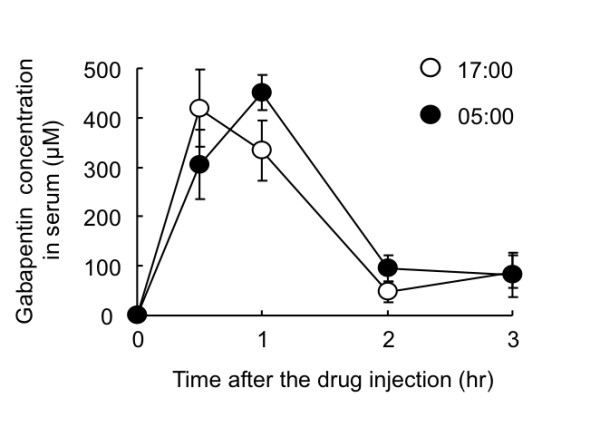
**Influence of dosing time on serum concentration of gabapentin in PSL mice**. Gabapentin (100 mg/kg) was administered i.p. at 17:00 or 5:00 on day 7 after nerve injury. Values are shown as means with S.E.M. (n = 5-6). Serum concentrations of gabapentin were measured by HPLC using fluorescence detection.

To determine the underlying mechanism of the dosing time-dependent difference in the anti-allodynic effects of gabapentin, we measured serum concentrations of gabapentin in PSL mice on day 7 after nerve injury. The serum levels peaked within 1 h after gabapentin injection (100 mg/kg, i.p.) and then biphasically decayed (Figure 3). However, the serum gabapentin concentration did not significantly differ between the two dosing times.

**Figure 4:**
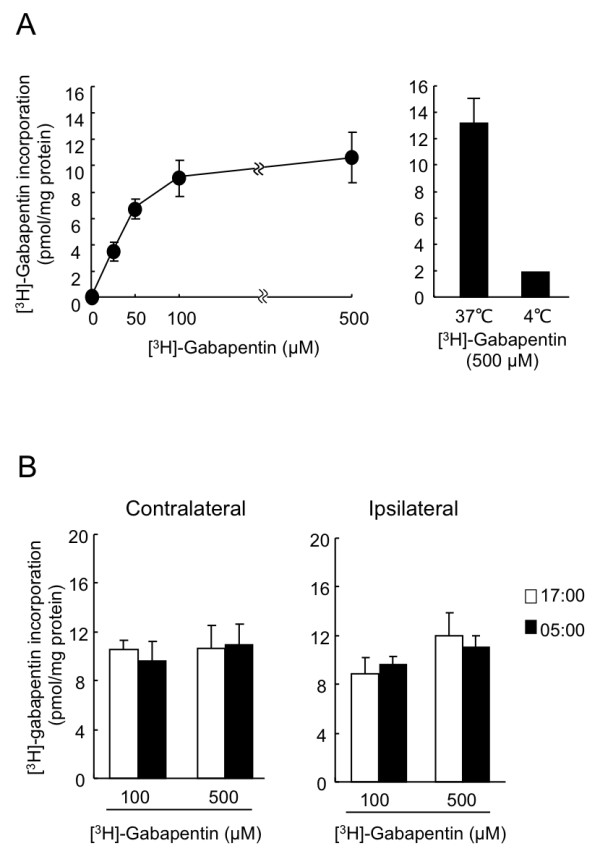
**Incorporation of [^3^H]-gabapentin into DRG of PSL mice is not changed time dependently**. (A) Left and right panels show concentration and temperature dependence of [^3^H]-gabapentin incorporation by ipsilateral DRG, respectively. Segments of ipsilateral DRG prepared from PLS mice were incubated for 30 min at 37°C or 4°C with Krebs-Henseleit buffer containing indicated concentrations of [^3^H]-gabapentin. Values are shown as means with S.E.M. (n = 3). (B) Contralateral (left) and ipsilateral (right) DRG prepared from PLS mice at 17:00 or 5:00 were incubated with Krebs-Henseleit buffer containing indicated concentrations of [^3^H]-gabapentin for 30 min at 37°C. Each value represents the mean with S.E.M. (n = 3).

Gabapentin is incorporated into cells via the L-amino acid transporter and it acts on the α2δ-1 subunit of VDCC to modulate Ca^2+ ^currents [12]. Because the L-amino acid transporter is expressed in the DRG neuron (Additional file 2), we further investigated whether gabapentin incorporation into L4/L5 DRG varied time-dependently. On day 7 after nerve injury, contralateral or ipsilateral DRG isolated from PSL mice were incubated with various concentrations of [^3^H]-gabapentin for 30 min. The incorporation of [^3^H]-gabapentin into the isolated DRG was concentration-dependent when the segments were incubated with the drug at 37°C (Figure 4A left panel). This process seemed to be energy-dependent because the amount of [^3^H]-gabapentin incorporation was significantly decreased by incubating the DRG at 4°C (Figure 4A right panel). However, the amount of [^3^H]-gabapentin incorporated into both the contralateral and ipsilateral DRG did not significantly differ between the two dosing times when the segments were incubated with 100 or 500 μM gabapentin (Figure 4B).

### Time-dependency of [^3^H]-gabapentin binding in the ipsilateral DRG

**Figure 5:**
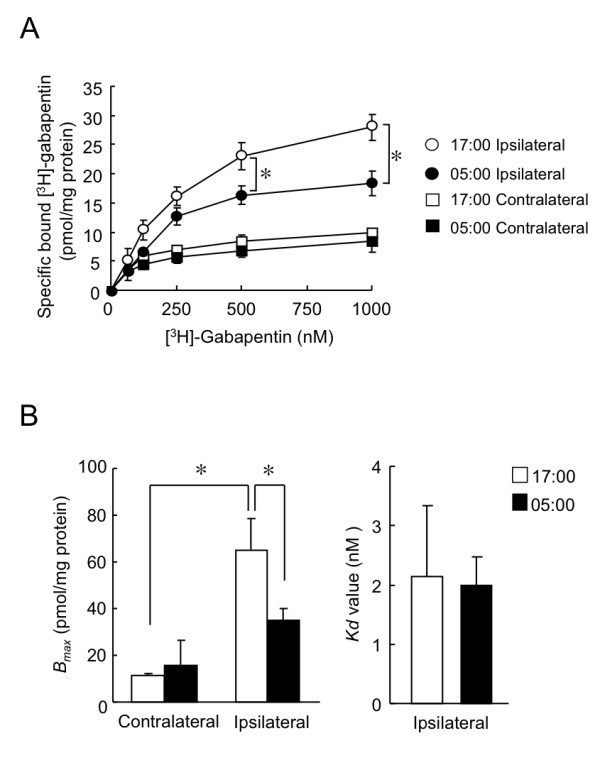
**Time-dependent [^3^H]-gabapentin binding in DRG of PSL mice**. (A) Concentration dependence of [^3^H]-gabapentin binding to soluble fraction of contralateral and ipsilateral DRG. Segments of DRG were prepared at 17:00 and 5:00. Values are shown as means with S.E.M. (n = 3). (B) Estimated maximum binding capacity (*B_max_*) of [^3^H]-gabapentin to soluble fraction of DRG of PSL mice and its affinity constants (*K_d_*). Values are shown as means with S.E.M. (n = 3). *P < 0.05 compared between two groups.

On day 7 after nerve injury, we estimated the binding parameters of gabapentin in L4/L5 DRG of PSL mice. The amount of specific binding of [^3^H]-gabapentin (*B_specific_*) in the ipsilateral DRG was larger than that in the contralateral DRG. Furthermore, the specific binding of [^3^H]-gabapentin in the ipsilateral DRG significantly varied in a time-dependent manner (P < 0.05; Figure 5A); a large amount of [^3^H]-gabapentin bound at 17:00. In contrast, the amount of [^3^H]-gabapentin binding in the contralateral DRG did not significantly vary time- dependently. Scatchard plot analysis revealed that the maximal binding capacity (*B_max_*) for [^3^H]-gabapentin in the ipsilateral DRG was significantly larger at 17:00 than at 5:00 (p < 0.05; Figure 5B left panel), whereas the affinity constants (*K_d_*) value did not differ significantly between ipsilateral DRG prepared at 17:00 and 5:00 (Figure 5B right panel).

### Time-dependent oscillation in the expression of α2δ-1 subunit in the ipsilateral DRG

**Figure 6:**
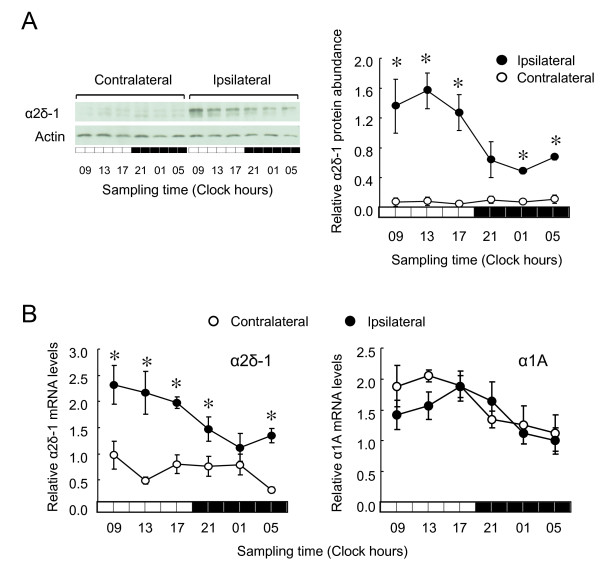
**Expression of α2δ-1 in DRG of PSL mice varies over 24 h**. (A) Temporal expression profile for α2δ-1 protein in contralateral and ipsilateral DRG of PSL mice. Values are normalized by protein levels of ACTIN. Values are shown as means with S.E.M. (n = 3). *P < 0.05 compared with contralateral side at corresponding time points. Levels of α2δ-1 protein significantly varied over 24 h in ipsilateral DRG (P < 0.05; ANOVA). Left panel shows representative western blots of α2δ-1 protein in contralateral and ipsilateral DRG of PSL mice. (B) Temporal expression profile of α2δ-1 (left) and α1A (right) mRNA in contralateral and ipsilateral DRG of PSL mice. Values are shown as means with S.E.M. (n = 4-6). *P < 0.05 compared with contralateral side at corresponding time points. Levels of α2δ-1 mRNA significantly varied over 24 h in ipsilateral DRG (P < 0.05; ANOVA).

On day 7 after nerve ligation, protein levels of α2δ-1 subunit of VDCC in the ipsilateral DRG substantially increased in response to nerve injury, but the levels fluctuated rhythmically in a circadian fashion (Figure 6A). The rhythmic phase of α2δ-1 protein levels was almost identical to the oscillation of maximal [^3^H]-gabapentin binding capacity (*B_max_*) of the ipsilateral DRG (Figure 5B). The mRNA levels of the α2δ-1 subunit also increased in the ipsilateral DRG of PSL mice and significantly oscillated over 24 h (Figure 6B, left panel). By contrast, the mRNA levels of other pore-forming subunits of VDCC, namely the α1A subunit did not significantly vary over 24 h (Figure 6B, right panel).

Taken together, these results suggest that the maximal [^3^H]-gabapentin binding capacity (*B_max_*) in the ipsilateral DRG would be changed according to the oscillation of α2δ-1 protein abundance. There was no significant time-dependent variation in the gabapentin incorporation into the ipsilateral DRG (Figure 4B). If the same amount of gabapentin could bind to α2δ-1 subunit at 17:00 and at 5:00, the occupancy of gabapentin binding sites in α2δ-1 in the ipsilateral DRG of PSL mice would be 2-fold higher at 5:00 than at 17:00.

## Discussion

Because the risk and/or intensity of disease symptoms vary during the day, many drugs should not be equally potent at different administration times. For example, the frequency of asthmatic episodes increases from midnight until the early morning, because of night time increases in airway potency, bronchial responsiveness and the release of inflammatory factors [19]. Therefore, evening dosing with theophylline produces a significant improvement in nocturnal asthmatic symptoms [20]. Evening dosing with H_2 _receptor antagonists is more effective for treating peptic ulcers because gastric secretion increases from evening to night [21]. A significant time-dependent variation has been identified in rheumatic and/or osteoarthritic pain [22,23]. Furthermore, the intensity of pain in patients with diabetic neuropathy or post-herpetic neuralgia also varies over 24 h [24]. However, chronopharmacological strategy has not been applied to the treatment of patients with neuropathic pain. Here, we found that the PWT in PSL mice significantly varied over 24 h; allodynic behavior was more severe from the late light, to the early dark phase. Rhythmic changes in allodynic pain among PSL mice seemed to be associated with dosing time-dependent differences in the anti-allodynic effects of gabapentin.

Both the sensitivity of living organisms to drugs and their pharmacokinetics influence drug effectiveness [25,26]. An i.p. injection of 100 mg/kg into PSL mice at 17:00 or 5:00 did not elicit significant dosing time-dependent differences in the serum concentration of gabapentin. Gabapentin is well absorbed orally, circulates unbound to plasma protein and it is excreted mostly in an unaltered form into urine [13]. The L-amino acid transporter is responsible for the intracellular uptake of gabapentin [27], which allows influxed gabapentin to act on its target in neurons. However, gabapentin incorporation into the ipsilateral DRG neurons did not significantly differ between the two dosing times. This may be explained partly by the arrhythmic expression of L-amino acid transporter-1 gene in the ipsilateral DRG (Additional file 2). Taken together, these results suggest that gabapentin pharmacokinetics do not principally contribute to the dosing time-dependent changes in its anti-allodynic action.

Although several mechanisms have been suggested for the anti-allodynic effects of gabapentin, the α2δ subunit of VDCC comprise the most attractive candidates as targets. The expression of α2δ subunit is up-regulated in the spinal cord and DRG of gabapentin-sensitive, but not gabapentin-insensitive neuropathic pain models [8]. Four α2δ subunits (α2δ-1, α2δ-2, α2δ-3, and α2δ-4) have been identified [28,29]. Primary sequence comparisons suggest that α2δ-3 and α2δ-4 lack the gabapentin binding motifs characterized for α2δ-1 and α2δ-2 [28,30]. Gabapentin has higher affinity for α2δ-1, than α2δ-2 subunits [28,30]. The protein levels of α2δ-1 subunit in the ipsilateral DRG of PSL mice were increased after nerve injury, but rhythmically fluctuated in a time-dependent manner. The mRNA levels of α2δ-1 in the ipsilateral DRG also exhibited a circadian oscillation, and the rhythmic phase of α2δ-1 mRNA expression was similar to that of its protein abundance. The circadian clock genes, the molecular components of the clockwork, regulate the expression of their target molecules at the transcription level [25,26]. The mRNA levels of clock genes in the ipsilateral DRG of PSL mice oscillated in a circadian-dependent manner (data not shown), indicating that the ipsilateral DRG has a normal clock function. Consequently, the oscillation in the expression of α2δ-1 in PSL mice is generated at the transcriptional level, which is controlled by the molecular organization of the circadian clockwork. Protein levels of α2δ-1 subunit in the DRG significantly correlate with the duration of neuropathic pain induced by nerve injury [7,9]. In fact, transgenic mice overexpressing the α2δ-1 subunit are hypersensitive to mechanical and thermal stimuli [31]. Therefore, oscillation of α2δ-1 subunit expression in the ipsilateral DRG might underlie the time-dependent change in the allodynic behavior of PSL mice.

The anti-allodynic effect of gabapentin was attenuated at the times of the day when α2δ-1 subunit protein was abundant. The amount of [^3^H]-gabapentin binding in the DRG was correlated with the protein levels of α2δ-1 subunit (Additional file 3), suggesting that the time-dependent change in the specific binding of [^3^H]-gabapentin is due to the oscillation in the expression of α2δ-1 protein. Consequently, scatchard plot analysis also revealed that the maximal binding capacity of gabapentin (*B_max_*) in the ipsilateral DRG varied significantly in a time-dependent manner. The maximal binding capacity of gabapentin was about 2-fold higher at 17:00 than at 5:00. On the other hand, the amounts of gabapentin incorporation into the ipsilateral DRG did not significantly differ between the two dosing times. If the same amount of gabapentin bound to α2δ-1 protein at 17:00 and at 5:00, the occupancy of gabapentin binding sites in α2δ-1 protein at 5:00 would be estimated 2-fold higher than that at 17:00. Therefore, the anti-allodynic effects of gabapentin may be enhanced by injection with the drug at 05:00. This would account for the dosing time-dependent change in the gabapentin-induced anti-allodynic effects.

## Conclusions

The present findings in this animal model help to clarify how the anti-allodynic effects of gabapentin vary according to dosing time. The intensity of neuropathic pain also changes time-dependently in patients with diabetic neuropathy and post-herpetic neuralgia [24], indicating that an appropriate dosing regimen is needed to treat such chronic pain. Our results suggest that the therapeutic efficacy of gabapentin on neuropathic pain could be improved by optimizing the dosing schedule.

## Materials and methods

### Animals and treatments

Five-week-old male ICR mice (Charles River Japan) were housed in groups of 6 or 10 per cage in a light-controlled room (lights on from 07:00 to 19:00) at 24 ± 1°C and humidity of 60 ± 10% with food and water ad libitum. They were synchronized to the lighting conditions for two weeks before experiment. We prepared mouse models of PSL under sodium pentobarbital (40 mg/kg, i.p.) or diethyl ether anesthesia. The left thigh was shaved and the sciatic nerve was exposed through an incision. Half of the nerve was tightly ligated with 8-0 silk thread and the wound was sutured. The left sciatic nerve was exposed and then the wound was closed without ligation in control, sham-operated mice. Animals were treated in accordance with the guidelines stipulated by the animal care and use committee of Kyushu University. Gabapentin (Tokyo Chemical Industry Co. Ltd., Tokyo, Japan) was dissolved in saline and injected i.p. in a volume of 0.1 mL/10 g body weight.

### Assessment of tactile allodynia

Mechanical allodynia was assessed using a dynamic plantar aesthesiometer (Ugo Basile, Varese, Italy), which is an automated von Frey-type system. The mice were placed in plastic cages with a wire mesh floor and allowed to acclimate for 15 min before measuring hind paw mechanical thresholds. A paw-flick response was elicited by applying increasing force (in grams) using a metallic filament focused on the middle of the plantar surface of the hind paw. The applied force was initially below the detection threshold, increased from 1 to 15 g in 0.5 g steps over 20 sec, and then maintained at 15 g for an additional 10 sec. The force applied to elicit a reflex removal of the hind paw was monitored. To evaluate the alleviation effects of gabapentin on tactile allodynia, the AUC was calculated using the trapezoidal rule over the entire time course of the PWT of the injured paw.

### Determination of gabapentin concentration in serum

The serum concentration of gabapentin was measured by high performance liquid chromatography (HPLC) using DL-norvaline (Wako, Osaka, Japan) as the internal standard. Gabapentin and DL-norvaline were derivatized with o-phthaldialdehyde (OPA; Wako) as described [32]. The mobile phase of phosphate buffer (pH 3.0; 20 mM)-acetonitrile (3:2, v/v) was eluted at 1.5 mL/min through a 5C_18_-MS-II column (4.6 × 150 mm; Nakalai Tesque, Kyoto, Japan) using an LC-20AD (Shimadzu, Kyoto, Japan), The separated analyte was detected using a Spectrofluorometric Detector RF-550 (excitation and emission at 330 and 450 nm, respectively; Shimadzu). The total separation lasted 30 min, with DL-norvaline and gabapentin eluting at 6 and 22 min, respectively.

### Determination of gabapentin incorporation into DRG

The L4/L5 DRG were isolated from PSL mice and pre-incubated for 15 min at 4°C or 37°C with 1 mL of Krebs-Henseleit buffer (118 mM NaCl, 4.8 mM KCl, 25 mM NaHCO_3_, 1.2 mM KH_2_PO_4_, 1.3 mM CaCl_2 _2H_2_O, 1.2 mM MgSO_4 _7H_2_O, 11 mM glucose, 0.06 mM ascorbic acid, 0.03 mM disodium EDTA 2H_2_O) and equilibrated with 95% O_2 _and 5% CO_2_. After pre-incubation for 20 min, [^3^H]-gabapentin (Muromachi Chemical Inc., Tokyo, Japan) was added to the incubation medium to a final concentration of 25, 50, 100, and 500 μM. The segments were incubated for 30 min. After incubation, the segments were rinsed three times with ice-cold PBS and homogenized with CelLytic^™ ^MT Cell Lysis Reagent (Sigma-Aldrich Company, Dorset, UK). The protein concentration was determined in half volumes of homogenates using Lowry's method (DC protein assay; Bio-Rad, HercuIes, CA). The radioactivity in the other half of the homogenate was solubilized and then determined using a liquid scintillation counter. The amounts of [^3^H]-gabapentin incorporated into the DRG are expressed as pmol/mg protein.

### Ultrafiltration binding assays

The L4/L5 DRG removed from 14 mice was pooled to obtain an adequate amount of protein and homogenized in 0.1 mL of ice-cold CeILytic™MT Cell Lysis Reagent (Sigma-Aldrich). The homogenate was then centrifuged at 4,000 rpm for 3 min at 4°C and the protein concentration was adjusted to 0.1 mg/mL. Binding was assayed in the soluble fraction containing 25 μg of DRG protein, and from 31 to 500 nM [^3^H]-gabapentin. Nonspecific binding was determined by adding a 100-fold excess of non-radiolabeled gabapentin. The total drug concentration was measured after incubation at 37°C for 30 min in 50 μL portions of each reaction mixture. A second aliquot (450 μL) of the mixture was filtered through an ultrafiltration membrane with a 50 kDa molecular weight cut-off (Millipore, Bedford, MA, USA). The amount of bound [^3^H]-gabapentin was determined by scintillation counting. Specific binding in the DRG was estimated as the ratio of [^3^H]-gabapentin drug concentrations in the ultrafiltrate and reaction mixture. Binding parameters, affinity constants (*Kd*) and maximum binding capacity (*B_max_*; pmol/mg protein) were estimated from three independent experiments.

### Western Blotting

The L4/L5 DRG removed from PSL mice at 09:00, 13:00, 17:00, 21:00, 01:00 and 5:00 were homogenized in CelLytic^™ ^MT Cell Lysis Reagent (Sigma-Aldrich). After centrifugation at 12,000 g for 15 min, proteins in the the supernatant (5 μg) were separated on sodium dodecyl sulfate-polyacrylamide gels, and transferred to a polyvinylidene difluoride membrane. The proteins on the membrane were reacted with antibodies against the α2δ-1 subunit (Alomone Labs, Jerusalem, Israel), or ACTIN (Santa Cruz Biotechnology, Santa Cruz, CA, USA). Specific antigen-antibody complexes were visualized using horseradish peroxidase-conjugated secondary antibodies and Chemi-Lumi One (Nakalai Tesque Inc., Kyoto, Japan).

### RT-PCR

Levels of the α1A and α2δ-1 subunits, as well as of β-actin mRNA were measured using real-time or semi-quantitative RT-PCR. Total RNA was extracted from L4/L5 DRG using RNAiso (Takara Bio Inc., Shiga, Japan) at the six time points described above. Complementary DNA (cDNA) was prepared via reverse transcription of total RNA using a ReverTra Ace^® ^qPCR RT kit (Toyobo, Osaka, Japan). Diluted cDNA samples were analyzed by Real-time PCR using THUNDERBIRDTM SYBR^® ^qPCR Mix (Toyobo) and the 7500 Real-time PCR system (Applied Biosystems, Foster City, CA). The sequences of the forward/reverse primers were as follows: α1A subunit, 5'-GCGAAGGCGACGATGGGGAG-3'/5'-CTGTGGCCCAGGCTGTCGTG-3'; α2δ-1 subunit, 5'-CGCGAAGATGGCTGCTGGCT-3'/5'-GGCGTGCATTGTTGGGCTCC-3'; β-actin, 5'-GACGGCCAGGTCATCACTATT-3'/5'-TACCACCAGACAGCACTGTGT-3'.

### Statistical analysis

The 0.05 level of probability was taken as the criterion for significance. The statistical significance of differences was analyzed by ANOVA and Fisher's PLSD test among multiple groups and by Student's t-test between pairs of groups.

## Aabbreviations

**DRG**: dorsal root ganglion; **GABA**: gamma-aminobutyric acid; **NSAID**: non-steroidal antiinflammatory drug; **PSL**: partial sciatic nerve ligation; **VDCC**: voltage-dependent Ca^2+ ^channels.

## Competing interests

The authors declare that they have no competing interests.

## Authors' contributions

NK and SK participated in the design of the study, conducted the experiments, analyzed the data, and drafted the manuscript. KH conducted the experiments. NM, MY, TU, MT, KI participated in the discussion of the experimental results and suggested the experiments. SO conceived the study, participated in its design and coordination, and wrote the manuscript. All authors read and approved the final manuscript.

## Supplementary Material

Additional file 1 **Influence of dosing-time on sedation induced by gabapentin**. Gabapentin (100 mg/kg) or saline were administered i.p. at 17:00 or 05:00 on day 7 after nerve injury. Assessment of locomotor activities was carried out for 4 hr post-injection using a photobeam activity monitoring system (Chronobiology Kit; Stanford Software Systems, California). Each value represents the mean with S.E.M. (n = 6). Click here for file

Additional file 2 **Temporal expression profile for L-amino acid transpotor-1 (LAT1) mRNA in DRG of PSL mice**. Each value represents the mean with S.E.M. (n = 4-6). Click here for file

Additional file 3 **The amount of gabapentin binding in the DRG is correlated with the protein levels of α2δ-1 subunit**. Upper panel shows the difference in the protein levels of α2δ-1 subunit in the lysates prepared from DRG. Lower panel shows the amount of gabapentin binding in the DRG lysates. Sample numbers correspond to that in upper panel. Click here for file
