# Supplementary figures and images for: Molecular basis for the dosing time-dependency of anti-allodynic effects of gabapentin in a mouse model of neuropathic pain
Source: Mol Pain. 2010 Nov 26;6:83. doi: 10.1186/1744-8069-6-83 (PMC3009974; doi:10.1186/1744-8069-6-83)

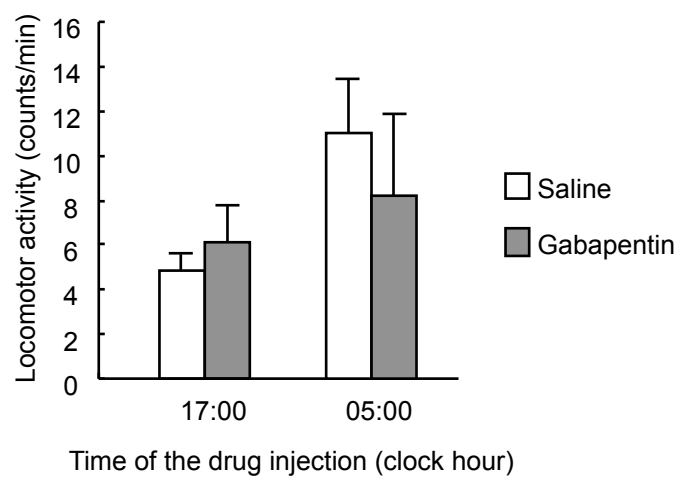

Supplement: Additional file 1 — Influence of dosing-time on sedation induced by gabapentin. Gabapentin (100 mg/kg) or saline were administered i.p. at 17:00 or 05:00 on day 7 after nerve injury. Assessment of locomotor activities was carried out for 4 hr post-injection using a photobeam activity monitoring system (Chronobiology Kit; Stanford Software Systems, California). Each value represents the mean with S.E.M. (n = 6). [file 1744-8069-6-83-S1.PDF]

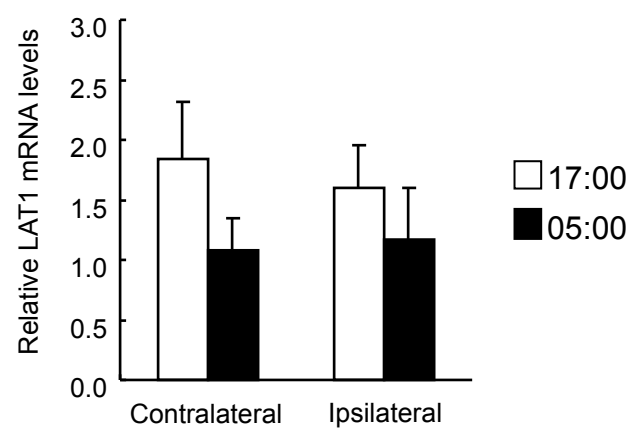

Supplement: Additional file 2 — Temporal expression profile for L-amino acid transpotor-1 (LAT1) mRNA in DRG of PSL mice. Each value represents the mean with S.E.M. (n = 4-6). [file 1744-8069-6-83-S2.PDF]

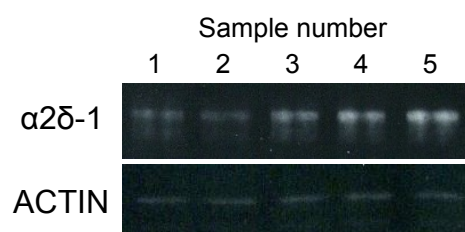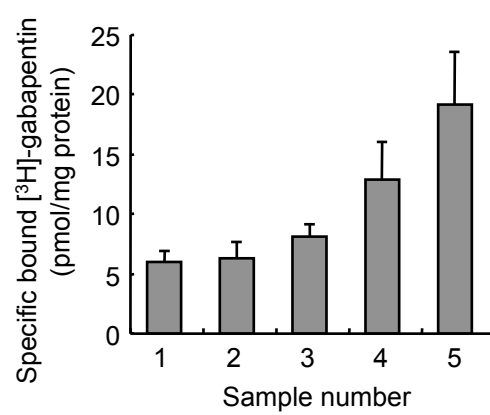

Supplement: Additional file 3 — The amount of gabapentin binding in the DRG is correlated with the protein levels of α2δ-1 subunit. Upper panel shows the difference in the protein levels of α2δ-1 subunit in the lysates prepared from DRG. Lower panel shows the amount of gabapentin binding in the DRG lysates. Sample numbers correspond to that in upper panel. [file 1744-8069-6-83-S3.PDF]
